# Low Serum Levels of (Dihydro-)Ceramides Reflect Liver Graft Dysfunction in a Real-World Cohort of Patients Post Liver Transplantation

**DOI:** 10.3390/ijms19040991

**Published:** 2018-03-26

**Authors:** Victoria Therese Mücke, Janis Gerharz, Katja Jakobi, Dominique Thomas, Nerea Ferreirós Bouzas, Marcus Maximilian Mücke, Sven Trötschler, Nina Weiler, Martin-Walter Welker, Stefan Zeuzem, Josef Pfeilschifter, Georgios Grammatikos

**Affiliations:** 1 Universitätsklinikum Frankfurt, Medizinische Klinik 1, Theodor-Stern-Kai 7, 60590 Frankfurt am Main, Germany; victoria.muecke@kgu.de (V.T.M.); Janisgerharz@web.de (J.G.); marcus.muecke@kgu.de (M.M.M.); sven.troetschler@kgu.de (S.T.); nina.weiler@kgu.de (N.W.); martin-walter.welker@kgu.de (M.-W.W.); zeuzem@em.uni-frankfurt.de (S.Z.); 2 Pharmazentrum Frankfurt, Institut für Allgemeine Pharmakologie und Toxikologie, Theodor-Stern-Kai 7, 60590 Frankfurt am Main, Germany; jakobi@med.uni-frankfurt.de (K.J.); pfeilschifter@em.uni-frankfurt.de (J.P.); 3 Institut für Klinische Pharmakologie und Toxikologie, Theodor-Stern-Kai 7, 60590 Frankfurt am Main, Germany; thomas@med.uni-frankfurt.de (D.T.); ferreirosbouzas@em.uni-frankfurt.de (N.F.B.)

**Keywords:** sphingolipids, orthopic liver transplantation, graft rejection, ischemic type biliary lesions

## Abstract

Patients after orthopic liver transplantation (OLT) are at risk of developing graft dysfunction. Sphingolipids (SL’s) have been identified to play a pivotal role in the regulation of hepatocellular apoptosis, inflammation and immunity. We aimed to investigate the serum SL profile in a prospective real-world cohort of post-OLT patients. From October 2015 until July 2016, 149 well-characterized post-OLT patients were analyzed. SL’s were assessed in serum probes via Liquid Chromatography/Tandem Mass Spectrometry. Twenty-nine (20%) patients had a biopsy proven graft rejection with decreased C20-ceramide (Cer) (*p* = 0.042), C18-dihydroceramide (DHC) (*p* = 0.022) and C24DHC (*p* = 0.060) levels. Furthermore, C18DHC (*p* = 0.044) and C24DHC (*p* = 0.011) were significantly down-regulated in patients with ischemic type biliary lesions (ITBL; *n* = 15; 10%). One-hundred and thirty-three patients (89%) have so far received tacrolimus as the main immunosuppressive agent with observed elevations of C14Cer (*p* = 0.052), C18Cer (*p* = 0.049) and C18:1Cer (*p* = 0.024). Hepatocellular carcinoma (HCC) pre-OLT was associated with increases in C24:1Cer (*p* = 0.024) and C24:1DHC (*p* = 0.024). In this large prospective cross-sectional study of patients, post-OLT serum levels of (very-)long chain (dihydro-)ceramides associate with graft rejection, ITBL, tacrolimus intake and HCC pre-OLT. Hence, serum SL’s may be indicative of graft complications. Further research is necessary to identify their diverse mechanistic role in regulating immunity and inflammation in patients post-OLT.

## 1. Introduction

In patients with end-stage liver disease, acute liver failure or selected cases of hepatocellular carcinoma (HCC), orthotopic liver transplantation (OLT) is often the last and sole available curative treatment [1,2,3]. Since the first successful OLT in 1967 [4] numerous aspects of pre- and post-OLT management have been improved, especially in terms of graft selection, surgeons’ techniques, ameliorated immunosuppressive therapy and early detection of complications [5]. Five-year survival rates of liver graft recipients have reached 60–95% depending on morphometric age and underlying disease [6,7,8]. Currently, immunosuppressive regimens based on calcineurin inhibitors (e.g., tacrolimus) constitute standard immunosuppressive therapy in most post-OLT patients [9,10]. However, liver graft rejections still remain a relevant clinical issue. Acute forms of graft rejections (such as portal hepatitis, endotheliitis and lymphocytic cholangitis) can be distinguished from chronic irreversible graft dysfunction (e.g., progressive duct loss and lipid-rich vasculopathy [11]).

In this context, Pfitzmann et al. showed in a large long-term outcome analysis that of post-OLT patients, 43.1% experienced at least one acute rejection and 3.5% at least one chronic graft rejection [12]. Counterbalanced by serious adverse effects of continuous immunosuppression, several authors discussed progressive reduction or even discontinuation of immunosuppressive therapy [13,14]. Hence, a better understanding of the underlying mechanisms and more accurate biomarkers predicting immune tolerance and graft rejection are urgently needed.

Recently, sphingolipids (SL’s) have been recognized as essential components and mediators of cellular apoptosis, proliferation and immune responses in liver injury, repair and regeneration [15]. Our group has already identified several serum SL parameters associated with different hepatopathies such as viral hepatitis, non-alcoholic fatty liver disease, decompensated liver cirrhosis and HCC [16,17,18]. Others revealed that in particular, ceramides (Cer’s) play a pivotal role in immune responses and prime adaptive immunity [19,20], while the question whether Cer related autophagy contributes to immunity or inflammation remains unanswered [21]. Many different stress stimuli seem to alter Cer synthesis and can influence its whole-body metabolism [22]. Moreover, Cer is an important mediator especially in liver regeneration. Consequently, Zabielski et al. demonstrated an increased concentration of Cer’s after partial hepatectomy in rats during liver regeneration [23]. Within de novo Cer-synthesis dihydro-ceramides (DHC’s) are immediate precursor molecules of Cer’s [24]. Lately, multiple investigators also uncovered DHC’s biological functions in autophagy, hypoxia and cellular proliferation [25]. The purpose of the current study was to investigate the predictive role of bioactive SL metabolites, especially DHC’s and Cer’s with distinct chain length, in a prospective real-world cohort of post-OLT patients, with regards to past or ongoing graft dysfunction. We intended to assess the potential diagnostic accuracy of serum SL’s as novel biomarkers in patients with graft rejection and with post-OLT complications such as ischemic type biliary lesions (ITBL).

## 2. Results

### 2.1. Patients’ Characteristics

Various SL parameters were assessed in serum probes of 149 post-OLT patients. Median age was 58 years (range 21–79), 55 (37%) were female, 94 (63%) were male. Represented pre-OLT liver diseases were viral hepatitis (hepatitis C virus(HCV) 23%, hepatitis B virus (HBV) 12%, HBV/hepatitis D virus (HDV)-coinfection 3%, HBV/HCV-coinfection 4.7%) and alcohol induced liver disease (23%). Less represented were primary biliary cholangitis (2.7%) and primary sclerosing cholangitis (3.6%). In 17 patients (11%), pre-OLT liver disease stayed cryptogenic. Almost one-third (*n* = 43, 29%) were transplanted due to HCC fulfilling Milan’s criteria [26]. Median graft liver age at study inclusion was 62.5 years (range 17–89), including 50 female donors (34%) and 62 male donors (42%; unknown donor gender was *n* = 37, 25%). In the majority of patients (*n* = 133, 89%), tacrolimus was part of current immunosuppressive therapy. Other mono- and combination-regimes included mycophenolate mofetil (*n* = 79, 53%), steroids (*n* = 16, 11%), mechanistic target of rapamycin-inhibitors (*n* = 12, 8%), cyclosporine A (*n* = 8, 5%) and basiliximab (*n* = 2, 1%). Overall, in 29 patients (20%), graft rejection was documented and proven by biopsy. Fifteen of all patients (10%) evolved ITBL, among them 5 (33%) who were biopsied. One-hundred and nine patients (73%) were positive for cytomegalovirus (CMV) IgG-antibodies. Further detailed patients’ characteristics and laboratory results are depicted in Table 1.

### 2.2. Low Serum Long and Very Long Chain (Dihydro-) Ceramides Associate with History of Graft Rejection and Ischemic Type Biliary Lesions (ITBL)

**Figure 1:**
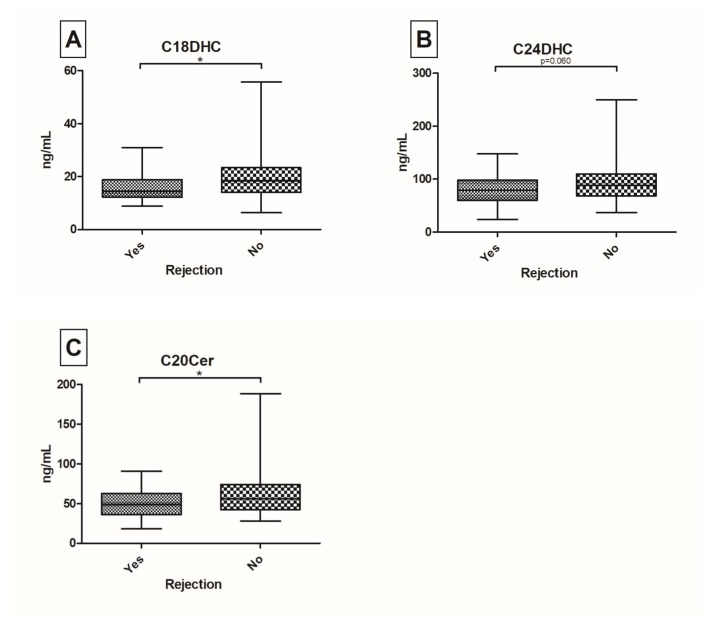
Serum ceramides (Cer) and dihydro-ceramides (DHC) in post-OLT patients with history of graft rejection. C18DHC (**A**), C24DHC (**B**) and C20Cer (**C**) are down-regulated in patients with graft rejection (* *p* < 0.05).

**Figure 2:**
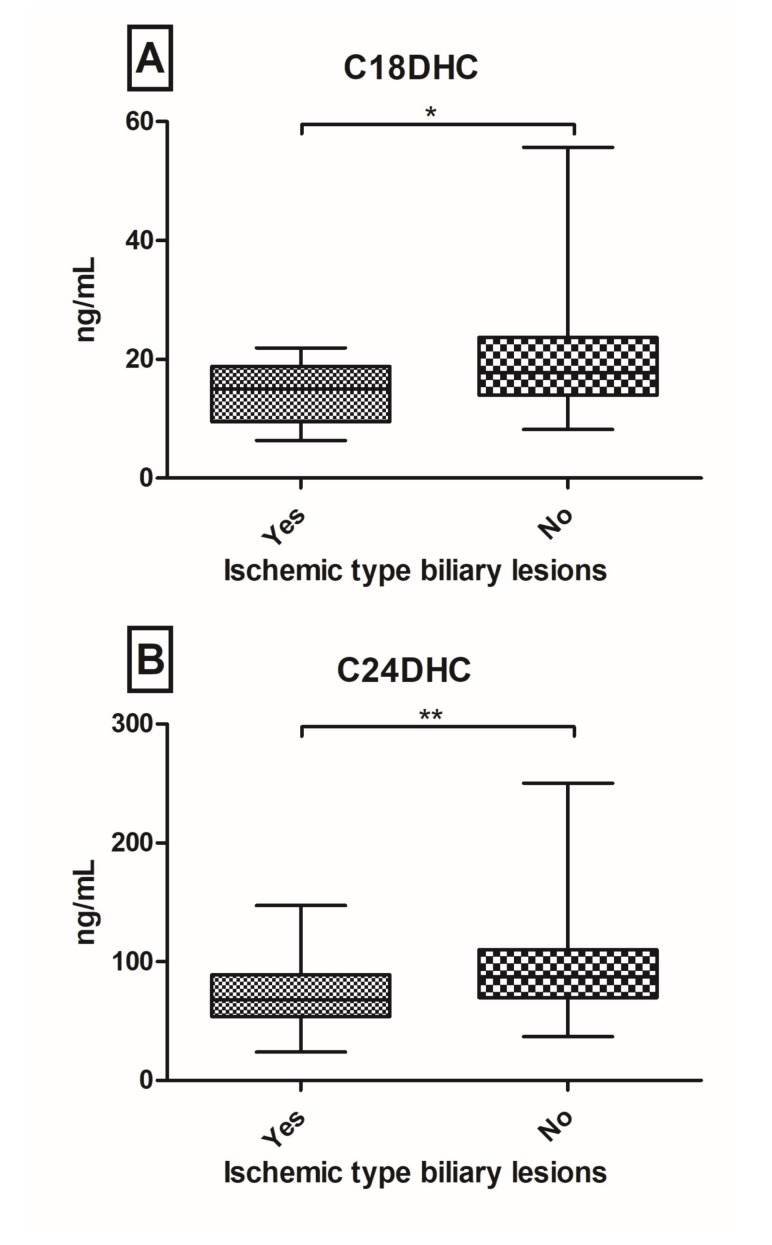
Serum dihydro-ceramides (DHC) in post-OLT patients with history of ischemic type biliary lesions. C18DHC (**A**) and C24DHC (**B**) are significantly down-regulated in patients with ischemic type biliary lesions (* *p* < 0.05; ** *p* < 0.01).

In our cohort, the highest serum concentrations were measured for C24Cer and C24:1Cer compared to C16Cer, C18Cer, C18:1Cer and C20Cer (mean levels of 1717 and 589 ng/mL versus 98, 48, 14, 54 ng/mL, respectively). Mean DHC levels were 19 ng/mL (C16DHC), 17 ng/mL (C18DHC), 86 ng/mL (C24DHC) and 64 ng/mL (C24:1DHC). Patients with a history of liver graft rejection had decreased serum levels of C20Cer (*p* = 0.042), C18DHC (*p* = 0.022) and C24DHC (*p* = 0.060) (Figure 1). In comparison, no significant changes were seen in serum levels of sphingosine, sphinganine, sphingosine-1-phosphate (S1P) or sphinganine-1-phosphate (dhS1P) between groups of graft rejection and no graft rejection (Supplementary Materials Figure S1). Both time between graft rejection and blood withdrawal and AB0-matching between liver donor and liver receiving patient were not associated with any significant alterations in SL parameters (*p* > 0.1) in univariate and multivariate analyses (Supplementary Materials Tables S1 and S2). Furthermore, C18DHC (*p* = 0.044) and C24DHC (*p* = 0.0095) were significantly down-regulated in patients with ITBL (Figure 2). Further SL parameters showed comparable median and ranges in ITBL versus non-ITBL patients (Supplementary Materials Figures S2 and S3). AST levels showed reciprocal correlations to C24Cer (*r* = −0.296, *p* < 0.001) and C20Cer (*r* = −0.215; *p* < 0.01), whereas ALT levels correlated positively to sphinganine (*r* = 0.194; *p* < 0.05), S1P (*r* = 0.171; *p* < 0.05), dhS1P (*r* = 0.211; *p* < 0.01) and C24:1DHC (*r* = 0.213; *p* < 0.01), and GGT to C16Cer (*r* = 0.164; *p* < 0.05), C18Cer (*r* = 0.203; *p* < 0.05) and C24:1DHC (*r* = 0.186; *p* < 0.05), respectively (Table 2). Given that serum SL levels were significantly associated with liver graft rejection and ITBL (Figure 1 and Figure 2), we included these parameters in a multivariate logistic regression model. In multivariate analysis, C18DHC (Odds-Ratio (*OR*) = 1.132; 95%-confidence interval (*CI*) = 1.009–1.269; *p* = 0.035) and C24Cer (*OR* = 1.001; *CI* = 1.0001–1.002; *p* = 0.039) were independently associated with graft rejection. In ITBL, besides C18DHC (*OR* = 1.172; *CI* = 1.006–1.366; *p* = 0.042), GGT (*OR* = 0.994, *CI* = 0.989–0.998; *p* = 0.006) showed significant associations after linear regression (Table 3).

### 2.3. Tacrolimus Based Immunosuppressive Therapy Associates with Upregulated Serum Cer Levels

**Figure 3:**
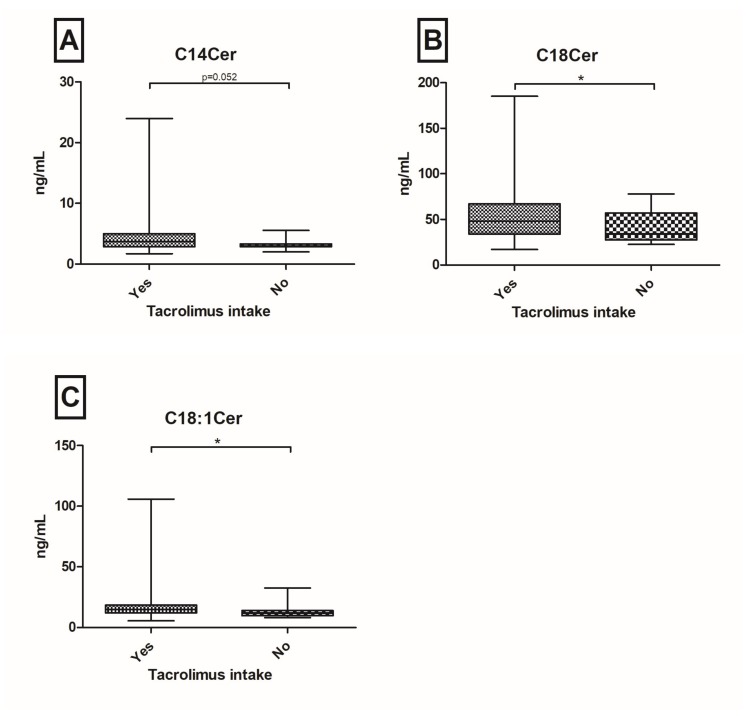
Serum ceramides (Cer) in post-OLT patients. C14Cer (**A**), C18Cer (**B**) and C18:1Cer (**C**) are up-regulated in patients with current tacrolimus intake, compared to no tacrolimus intake (* *p* < 0.05).

The majority of all included patients (*n* = 133, 89%) received tacrolimus as the base of their immunosuppressive therapy. Their serum concentrations of C14Cer (*p* = 0.052), C18Cer (*p* = 0.049) and C18:1Cer (*p* = 0.024) were up-regulated compared to patients without tacrolimus treatment (Figure 3). Due to the small number of patients who were not receiving tacrolimus (*n* = 16), we did supplementary analyses in propensity score matched patients. Three patients with tacrolimus intake were matched to one patient, who was not taking tacrolimus at the time of blood withdrawal (in relation 3:1). Variables for matching included age, gender, ALT, history of graft rejection and history of HCC pre-OLT. In this propensity score matched subgroup, C14Cer (*p* = 0.172) and C18Cer (*p* = 0.099) were no longer significantly up-regulated in patients with tacrolimus intake, but C18:1Cer (*p* = 0.017) was still significantly up-regulated (Supplementary Materials Figure S4). No significant correlations could be observed between tacrolimus trough levels and SL concentrations (Table 2). However, in multivariate analyses, tacrolimus through levels influenced the serum levels of long-chain ceramides (Supplementary Materials Tables S3–S5). Additionally, other immunosuppressive agents such as mycophenolate-mofetil, steroids, mechanistic target of rapamycin-inhibitors, cyclosporine A and basiliximab did not show significant SL alterations in our cohort; however, only a small number of patients received these regimes.

### 2.4. Analyses of Serum Cer’s Concerning Pre-OLT Hepatopathy, HCC, Donors’ Characteristics and Time Between OLT and Blood Withdrawal

**Figure 4:**
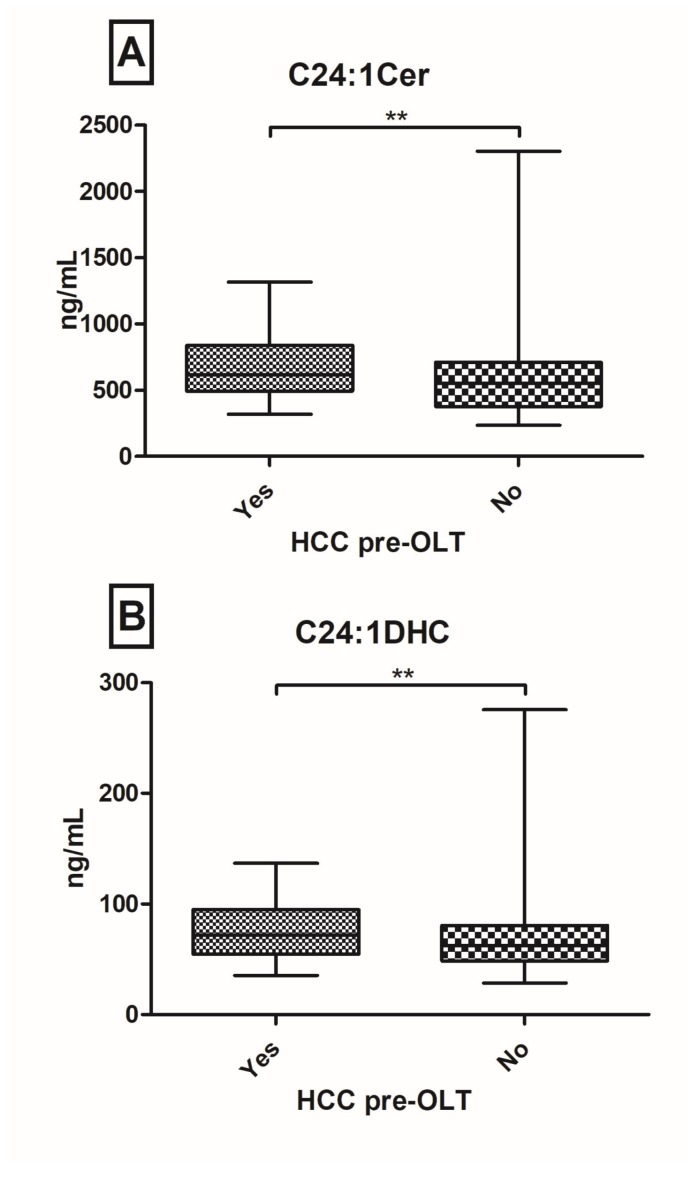
Serum ceramides (Cer) and dihydro-ceramides (DHC) in post-orthopic liver transplantation (OLT) patients with a history of hepatocellular carcinoma (HCC) pre-OLT. C24:1Cer (**A**) and C24:1DHC (**B**) are significantly up-regulated in patients with HCC pre-OLT, compared to no prior HCC (** *p* < 0.01).

In general, pre-OLT aetiology of hepatopathy did not show significant impact on post-OLT SL parameters. However, patients with HCC prior OLT showed significant SL alterations in our post-OLT cohort. C24:1Cer (*p* = 0.024) and C24:1DHC (*p* = 0.024) were significantly elevated in patients with pre-OLT HCC compared to patients without pre-OLT HCC (Figure 4). Of note, there were no signs of HCC recurrence at the date of inclusion. Unfortunately, multivariate analyses including different clinical and biochemical parameters could not show independent influence of HCC pre-OLT on C24:1Cer and C24:1DHC (Supplementary Materials Tables S4 and S5).

Concerning donor’s characteristics, we could observe decreased S1P-levels in female donors compared to male donors (*p* = 0.049), decreased S1P levels in CMV positive liver grafts (*p* = 0.024, missing data in 43 cases) and a negative correlation between graft age and C24:1Cer (*r* = 0.197; *p* < 0.05) (Table 2).

Median time between last OLT and study inclusion was 117 months (range 1–382 months). Serum levels of C16Cer, C20Cer, C24Cer and C24:1Cer showed significant reciprocal correlations with this timespan (Table 2). Moreover, SL concentrations were comparable between patients with or without CMV IgG antibodies (*p* > 0.05, missing data *n* = 4). No significant alterations were seen in SL profiles in patients with or without hepatic artery thrombosis (HAT, missing data *n* = 23) after OLT (*p* > 0.05).

## 3. Discussion

In this study, we analyzed SL profiles of patients post-OLT in a German tertiary liver transplant centre in detail. We report down-regulated serum Cer and DHC levels in association with graft dysfunctions, especially graft rejection, hepatic inflammation and ITBL. Immunosuppressive therapies including tacrolimus seem to elevate serum ceramide levels. Furthermore, pre-OLT HCC is associated with elevated Cer and DHC parameters in our post-OLT cohort. To the best of our knowledge, we are the first to describe these correlations, which may influence future diagnostic and therapeutic strategies in patients post-OLT.

Previously, we identified serum SL’s as potential biomarkers in hepatopathies. In particular, serum-Cer concentrations have been associated with the severity of liver cirrhosis and the occurrence of hepatic decompensation, as well as overall survival [18] and HCC [17]. Our current study focused on associations between liver graft complications and serum SL’s. In this large prospective cross-sectional study of patients post-OLT, low serum levels of long chain Cer’s and (very-)long chain DHC’s -precursors of Cer’s- are associated with graft rejection and ITBL. Both univariate and multivariate analyses revealed that C24Cer and C18DHC are the sole independent predictors of graft rejection (Figure 1, Table 3). C18DHC, already significantly decreased in univariate analysis (Figure 2), and GGT, mainly representing biliary impairment, are the sole significant predictors of ITBL. These findings underline, at a translational level, the already described associations of SL signalling inflammatory activity reported by many authors: various chain lengths and saturations of the fatty acid result in different pathological impact of SL’s in autophagy, inflammation, apoptosis and cancer [27]. In vitro studies revealed that very-long chain Cer’s (≥C24Cer) and their dihydro-precursors (DHC’s) mainly promote proliferation [28,29], whereas shorter Cer’s (i.e., C16Cer) show pro-apoptotic effects [30,31]. Our observations of decreased (very-)long chain Cer’s and DHC’s reflect a tendency for pro-apoptotic metabolic states in liver graft complications and may contribute to long-term hepatic complications. In hepatic pathophysiology, massive apoptosis leads to an activation of hepatic stellate cells, which results in progressive liver fibrosis and in the long-term, in liver cirrhosis [32,33]. Conversely, we could observe that immunosuppressive therapy regimes including tacrolimus go along with elevated serum shorter chain ceramide concentrations (C14Cer, C18Cer and C18:1Cer). Hence, serum SL’s may be indicative of graft dysfunctions, inflammation and immune tolerance post-OLT, but further investigations will be needed to learn more about detailed roles and interacting pathophysiological mechanisms.

Furthermore, this cohort could confirm our previous observations of increased Cer levels in HCC patients [17]. After initially excluding three patients with active HCC recurrence in our analysis we could still observe that a history of pre-OLT HCC development was significantly associated with increased levels of C24:1Cer and C24:1DHC (*p* < 0.05). At the date of inclusion there were no signs of HCC recurrence in these patients. Our results would therefore suggest a more pro-proliferative state in these patients, while a prolonged longitudinal follow-up concerning SL profile and late HCC recurrence would be interesting. Nevertheless, these findings support SL’s’ potential as HCC biomarkers and may be useful in post-OLT patients.

Moreover, the therapeutic potential to modulate the balance between Cer, sphingosine and S1P has already been shown in several preclinical studies [34]: On the one hand, methods elevating cellular Cer levels have been used to arrest growth or promote cell apoptosis in cell cultures [35,36]. On the other hand, reducing cellular Cer levels or increasing S1P leads to proliferation and attenuation of apoptosis. Tamura et al. demonstrated that the S1P-analogon fingolimod (FTY720)—a functional S1P inhibitor—can reduce liver graft dysfunction in rat liver transplants [37]. Allografts that underwent combination therapy with FTY720 and tacrolimus showed markedly reduced lymphocyte infiltration. This synergistic therapy allowed lower tacrolimus doses, which could avoid associated long-term side effects in future. Similar observations were made by combining FYT720 with cyclosporine A in renal graft recipient rats and dogs. Graft survival was significantly prolonged [38]. In a mouse model of chronic vascular rejection, Trayssac et al. demonstrated a significant role of S1P in mitogenic signalling, which could be effectively influenced by inhibitors of the sphingosine kinase 1/S1P pathway [39]. Furthermore, Förster et al. showed that S1P mediates the inhibition of stress-induced mesangial-cell apoptosis of dexamethasone [40]. Thus, bioactive SL’s may support future immunosuppressive therapy regimes in transplantation or autoimmune disorders. So far, most studies in this context were focused on S1P. Even though sphingosines and Cer’s synthesis are closely linked, there are multiple different pathways in SL metabolism. It would be interesting to investigate manipulated Cer and DHC metabolism in similar scenarios with regard to our current clinical observations.

Despite our promising results, our study has several limitations. First, we obtained serum samples of our patients only once. Longitudinal analyses on fixed time points after OLT and on different acute inflammatory events would be of great interest. Second, due to patients’ data protection and multi-centre allocation, there are only limited data about liver donors’ characteristics such as comorbidities, family history or lifestyle. This information may influence our observations, as we could see slight differences in SL profile according to donor’s gender, CMV status and graft age. Third, according to current literature, further in vivo and in vitro studies are needed to identify direct correlations and associations between serum and cellular SL levels. Detailed understanding could open opportunities for more translational trials and possible therapeutic consequences.

In conclusion, in a European real-world cohort of post-OLT patients, we are the first to report associations between low serum DHC- and Cer-levels and liver graft dysfunction, such as graft rejection and ITBL. Current standard immunosuppressive therapy including tacrolimus may alter the Cer pathway and is significantly associated with elevated serum SL levels. Moreover, pre-OLT HCC without signs of post-OLT recurrence was associated with elevated DHC and Cer levels and may underline the role of SL’s as potential HCC biomarkers [17]. The complexity of SL pathways and still limited knowledge of the underlying mechanisms concerning immunity and inflammation leave great scope to discuss our current results. Further research will be required to identify the diverse mechanistic role of SL’s in regulating immunity and inflammation after liver transplantation, but a favourable predictive potential and therapeutic use can be assumed.

## 4. Patients and Methods

The present study was carried out in accordance with the Declaration of Helsinki and after approval of the local ethics committee (Ethik-Kommission des Fachbereichs Medizin der Goethe Universität Frankfurt am Main, number 268/13-007, date of approval 14 January 2016). Standards of good clinical practice were followed at all times during patients’ care and the conducting of the study. No donors’ organs were obtained from executed prisoners or other institutionalized persons.

### 4.1. Patients’ Selection

At the outpatient’s clinic of the Goethe University Hospital Frankfurt am Main, Germany, a large tertiary and liver transplant centre, patients post-OLT due to any liver disease were recruited for this study. All patients signed informed consent agreements to participate in the local liver transplant registry. Patients were included if age > 18 years, post-OLT due to any former liver disease, no solid organ transplantation other than liver and serum retention samples had been obtained. From October 2015 until July 2016, 152 patients were eligible. With regard to our previous studies concerning SL alterations in patients with HCC (16), three patients with active HCC recurrence post-OLT were primarily excluded from further analyses to avoid biased results of serologic SL profile due to HCC. Age, gender, aetiology of former liver disease, date of liver transplantation, CMV infection status, transaminases, liver synthesis, history of organ rejection and immunosuppressive therapy were raised or withdrawn from clinical charts. As previously described [41], the Banff schema was used to grade acute cellular rejection in liver biopsies [42]. Episodes of putative graft complications were primarily defined by biochemical parameters, such as increase in bilirubin, gamma glutamyl transferase (GGT), aspartate (AST) or alanine transferase (ALT). Further invasive liver biopsy was triggered in case of clinical suspected rejection. Additionally, liver donor information such as age at organ explantation, gender, CMV status and hepatitis B virus core antibody (HBcAb) was gathered. Blood samples were obtained between 8:00 a.m. and 2:00 p.m. Patients were expected to be fasting prior to blood analysis and ultrasound examination. Patients’ nutritional habits were not documented.

### 4.2. Determination of Sphingolipid Concentrations by High-Performance Liquid Chromatography Tandem Mass Spectrometry

For the quantitation of sphingolipids, 10 µL serum was mixed with 190 µL water, 200 µL extraction buffer (citric acid 30 mM, disodium hydrogen phosphate 40 mM) and 20 µL of the internal standard solution containing sphingosine-d7, sphinganine-d7 (200 ng/mL each), sphingosine-1-phosphate-d7, C17:0 Cer, C16:0 Cer-d31, C18:0 Cer-d3, C17:0 LacCer, C18:0 DHC-d3, C16:0 LacCer-d3, C18:0 GluCer-d5 (all avanti polar lipids, Alabaster, AL, USA) and C24:0 Cer-d4 (Chiroblock GmbH, Bitterfeld-Wolfen, Germany) (400 ng/mL methanol each). The mixture was extracted once with 1000 µL methanol/chloroform/hydrochloric acid (15:83:2, *v*/*v*/*v*). The lower organic phase was evaporated at 45 °C under a gentle stream of nitrogen and reconstituted in 100 µL of tetrahydrofuran/water (9:1, *v*/*v*) with 0.2 formic acid and 10 mM ammonium formate. Afterwards, amounts of the sphingolipids were analyzed by liquid chromatography coupled to tandem mass spectrometry (LC-MS/MS). An Agilent 1100 series binary pump (Agilent technologies, Waldbronn, Germany) equipped with a Luna C8 column (150 mm × 2 mm ID, 3 μm particle size, 100 Å pore size; Phenomenex, Aschaffenburg, Germany) was used for chromatographic separation. The column temperature was 35 °C. The HPLC mobile phases consisted of water with 0.2% formic acid and 2 mM ammonium formate (mobile phase A) and acetonitrile/isopropanol/acetone (50:30:20, *v*/*v*/*v*) with 0.2% formic acid (mobile phase B). For separation, a gradient program was used at a flow rate of 0.3 mL/min. The initial buffer composition 55% (A)/45% (B) was held for 0.7 min and then within 4.0 min, linearly changed to 0% (A)/100% (B) and held for 13.3 min. Subsequently, the composition was linearly changed within 1.0 min to 75% (A)/25% (B) and then held for another 2.0 min. The total running time was 21 min, and the injection volume was 15 μL. To improve ionization, acetonitrile with 0.1% formic acid was infused post-column using an isocratic pump at a flow rate of 0.15 mL/min. After every sample, sample solvent was injected for washing the column with a 12 min run. The MS/MS analyses were performed using a triple quadrupole mass spectrometer API4000 (Sciex, Darmstadt, Germany) equipped with a Turbo V Ion Source operating in positive electrospray ionization mode. The MS parameters were set as follows: Ionspray voltage 5500 V, source temperature 500 °C, curtain gas 30 psi, collision gas 12 psi, nebulizer gas 40 psi and heating gas 60 psi. The analysis was done in Multiple Reaction Monitoring (MRM) mode with a dwell time of 20 ms for all analytes. Precursor-to-product ion transitions used for quantification of sphingolipids and corresponding mass spectrometric parameters are given in Supplementary Materials Table S6.

Data acquisition was done using Analyst Software V 1.6 and quantification was performed with MultiQuant Software V 3.0 (both Sciex, Darmstadt, Germany), employing the internal standard method (isotope dilution mass spectrometry). GluCer and LacCer were automatically measured in standard internal solution mix but were not used in further analyses. Variations in the accuracy of the calibration standards were less than 15% over the whole range of calibration, except for the lower limit of quantification, where a variation in accuracy of 20% was accepted.

### 4.3. Statistical Analyses

Statistical calculations were performed by using BiAS software for Windows (version 11.05; Epsilon-Verlag, Darmstadt, Germany). Analysis for the presented box plots was performed with GraphPad Prism for Windows (v5.02; GraphPad Software Inc., San Diego, CA, USA). Differences between patients’ subgroups were calculated by the non-parametric Mann-Whitney U test or Kruskal-Wallis tests. To identify parameters independently associated with, e.g., graft rejection or ITBL, post univariate analysis, step-wise logistic regression analysis was performed with backward selection, using *p* value ≥ 0.1 for removal from the model. Correlation analyses were performed by Spearman’s rank correlation. Propensity score calculations were performed by using R software (R Core Team 2015, Vienna, Austria) and analysis program MatchIt by Ho, Imai, King and Stuart (2011). *p* values < 0.05 were considered as statistically significant and were depicted in corresponding figures by using asterixis: * *p* < 0.05, ** *p* < 0.01 and *** *p* < 0.001.

## Figures and Tables

**Table 1:** Patients’ characteristics.

Parameters	Patients (*n* = 149)
Age, years; mean (range)	56 (21–79)
Gender	
Female, *n* (%)	55 (37%)
Male, *n* (%)	94 (63%)
Body mass index; mean (range)	26 (16.4–46.5)
Blood type A/B/AB/0, *n* (%)	57 (38%)/23 (15%)/7 (4.6%)/39 (26%)
ALT, IU/L; mean (range)	30 (7–132)
AST, IU/L; mean (range)	30 (13–100)
GGT, IU/L; mean (range)	89 (8–1525)
Bilirubin, mg/dL; mean (range)	0.8 (0.1–19)
Creatinine, mg/dL; mean (range)	1.3 (0.64–9.76)
Triglyceride, mg/dL; mean (range)	134 (40–411)
Cholesterol, mg/dL; mean (range)	178 (38–331)
Albumin, mg/dL; mean (range)	4.3 (3–4.9)
International normalized ratio; mean (range)	1.07 (0.84–3.38)
Tacrolimus though level, ng/ml; mean (range) of 133 patients	5.9 (2.4–23.1)
Aetiology pre-OLT chronic liver disease	
Hepatitis B, *n* (%)	18 (12%)
Hepatitis B/D coinfection, *n* (%)	5 (3%)
Hepatitis C, *n* (%)	34 (22.8%)
Hepatitis B/C coinfection, *n* (%)	7 (4.7%)
Alcohol intake, *n* (%)	35 (23%)
Non-alcoholic steatohepatitis, *n* (%)	1 (0.7%)
Primary sclerosing cholangitis, *n* (%)	5 (3.6%)
Primary biliary cholangitis, *n* (%)	4 (2.7%)
Cryptogenic, *n* (%)	17 (11%)
Other, *n* (%)	32 (21.5%)
HCC pre-OLT, *n* (%)	43 (28.9%)
CMV-IgG-antibody positive, *n* (%)	109 (73%)
Graft rejection, *n* (%)	29 (19.5%)
Ischemic type biliary lesions, *n* (%)	15 (10%)
Immunosuppressive therapy	
Tacrolimus, *n* (%)	133 (89%)
Mycophenolate-mofetil, *n* (%)	79 (53%)
Cyclosporine A, *n* (%)	8 (5%)
mTor-Inhibitors, *n* (%)	12 (8%)
Steroids, *n* (%)	16 (10.7%)
Basiliximab, *n* (%)	2 (1%)
Donors’ gender	
Female, *n* (%)	50 (33.5%)
Male, *n* (%)	62 (41.6%)
Not applicable, *n* (%)	37 (24.8%)
Grafts’ age, years; mean (range)	58 (17–89)
AB0-matching: identical/compatible/incompatible, *n* (%)	114 (77%)/10 (6.7%)/0 (0%)

Mean with range or number of patients with percent in parentheses. Abbreviations: ALT, alanine transferase; AST, aspartate transferase; GGT, gamma glutamyl transferase; OLT, orthopic liver transplantation; HCC, hepatocellular carcinoma; CMV, cytomegalovirus; mTor, mechanistic target of rapamycin. Missing data: Blood types were missing for 23 patients; GGT levels were missing for 1 patient, CMV antibody state was missing for 4 patients, tacrolimus through levels were missing for 2 patients with current tacrolimus intake, age of graft was missing in 37 cases, AB0-matching was missing in 25 cases. Multiple assessments possible.

**Table 2:** Correlations of serum SL’s of patients post-OLT with age of patients and grafts, biochemical parameters and time between OLT and inclusion.

SL	Patients’ Age at BW	AST	ALT	GGT	Chol	TG	Tacrolimus Though Level	Grafts’ Age at BW	Time Between OLT and BW
Sphingosine	0.149	0.030	0.147	0.062	0.120	0.095	−0.0001	0.104	−00.031
Sphinganine	0.047	0.036	0.194 *	0.049	0.082	0.065	−0.039	0.114	−0.031
S1P	−0.014	0.069	0.171 *	0.059	0.225 **	0.114	0.027	−0.111	−0.014
dhS1P	−0.046	0.104	0.211 **	0.049	0.211 **	0.165 *	0.037	−0.040	0.030
C24Cer	0.214 **	−0.296 ***	−0.050	−0.145	0.606 ***	0.429 ***	0.159	−0.110	−0.186 *
C16Cer	0.059	0.065	0.016	0.164 *	0.205 **	0.124	−0.079	0.081	−0.199 *
C14Cer	0.069	0.037	0.125	0.101	0.284 ***	0.129	−0.014	−0.018	−0.096
C18Cer	0.308 ***	−0.029	0.115	0.203 *	0.427 ***	0.455 ***	−0.051	−0.086	−0.142
C20Cer	0.306 ***	−0.215 **	−0.071	0.060	0.396 ***	0.418 ***	0.007	−0.154	−0.167 *
C18:1Cer	0.031	−0.041	−0.0003	0.022	0.217 **	0.136	0.008	0.075	−0.094
C24:1Cer	0.291 ***	−0.153	−0.004	0.082	0.401 ***	0.444 ***	0.108	−0.197 *	−0.208 *
C16DHC	−0.010	0.142	0.076	0.137	0.039	0.040	−0.082	0.106	−0.072
C18DHC	0.273 ***	−0.034	0.078	0.142	0.284 ***	0.377 ***	−0.071	0.030	−0.093
C24DHC	0.161	−0.104	0.101	−0.055	0.529 ***	0.313 ***	0.062	0.001	−0.130
C24:1DHC	0.199 *	0.109	0.213 **	0.186 *	0.317 ***	0.272 ***	−0.063	0.033	−0.040

Correlations are evaluated by Spearman’s rank correlation coefficient rho (r). Significant correlations are indicated in the corresponding figures: * *p* < 0.05; ** *p* < 0.01; *** *p* < 0.001. Abbreviations: SL, sphingolipid; OLT, orthopic liver transplantation; AST, aspartate transferase; ALT, alanine transferase; GGT, gamma glutamyl transferase; Chol, cholesterol; TG, triglyceride; BW, blood withdrawal; S1P, sphingosine-1-phosphate; dhS1P, sphinganine-1-phosphate; Cer, ceramide; DHC, dihydroceramide. Missing data: GGT was missing for 1 patient, tacrolimus through levels were missing for 2 patients with current tacrolimus intake, missing age of graft in 37 cases.

**Table 3:** Multivariate analyses for history of liver graft rejection (A) and ischemic type biliary lesions (B).

	Univariate Analysis		Multivariate Analysis	
Variable	*p* Value	OR (95% CI)	*p* Value	OR (95% CI)
Age	0.919	0.997 (0.942–1.055)		
Gender patient	0.339	1.704 (0.572–5.081)		
Gender donor	0.938	0.959 (0.332–2.770)		
ITBL	0.388	1.902 (0.441–8.194)		
HCC pre-OLT	0.353	0.581 (0.185–1.828)		
Tacrolimus intake	0.207	0.296 (0.045–1.965)		
ALT	0.091	0.980 (0.957–1.003)		
AST	0.138	0.973 (0.938–1.009)		
GGT	0.892	1.000 (0.997–1.003)		
Sphingosine-1-phosphate	0.749	0.999 (0.993–1.005)		
Sphinganine	0.932	0.992 (0.823–1.195)		
Sphinganine-1-phosphate	0.449	0.993 (0.973–1.012)		
C18Cer	0.475	1.009 (0.985–1.033)	0.072	0.973 (0.945–1.002)
C18:1Cer	0.571	1.016 (0.962–1.072)		
C20Cer	0.225	1.017 (0.990–1.045)		
C24Cer	0.048	1.001 (1.000–1.002)	0.039	1.001 (1.000–1.002)
C18DHC	0.073	1.084 (0.992–1.183)	0.035	1.132 (1.009–1.269)
C24:1DHC	0.113	1.018 (0.994–1.043)		
Age	0.311	1.032 (0.971–1.097)		
Gender patient	0.292	2.000 (0.551–7.256)	0.083	3.718 (0.842–16.424)
Gender donor	0.863	0.894 (0.251–3.190)		
Graft rejection	0.388	1.902 (0.441–8.195)		
HCC pre-OLT	0.818	0.857 (0.230–3.189)		
Tacrolimus intake	0.933	2.741×10^4^ (0.000–3.39×10^107^)		
ALT	0.204	0.984 (0.959–1.009)		
AST	0.294	0.979 (0.941–1.019)		
GGT	0.003	0.994 (0.989–0.998)	0.006	0.994 (0.989–0.998)
C16Cer	0.931	1.001 (0.982–1.020)		
C18DHC	0.025	1.158 (1.019–1.316)	0.042	1.172 (1.006–1.366)
C24DHC	0.082	1.024 (0.997–1.052)		
C24:1DHC	0.375	1.012 (0.986–1.040)		

Abbreviations: OR, odds-ratio; CI, confidence interval; ITBL, ischemic type biliary lesions; HCC, hepatocellular carcinoma; OLT, orthopic liver transplantation; ALT, alanine transferase; AST, aspartate transferase; GGT, gamma glutamyl transferase; Cer, ceramide; DHC, dihydroceramide. Missing data: GGT level was missing for 1 patient, unknown donors’ gender for 37 patients; missing CMV state of donor in 43 cases, missing age of graft in 37 cases.

## Supplementary Materials

The following are available online at http://www.mdpi.com/1422-0067/19/4/991/s1.
