# Supplementary material for: Low Serum Levels of (Dihydro-)Ceramides Reflect Liver Graft Dysfunction in a Real-World Cohort of Patients Post Liver Transplantation
Source: Int J Mol Sci. 2018 Mar 26;19(4):991. doi: 10.3390/ijms19040991 (PMC5979454; doi:10.3390/ijms19040991)
Supplement: Supplementary file 1 [file ijms-19-00991-s001.pdf]

Supplementary information

Suppl. Table S1. Multivariate analysis for history of liver graft rejection including time between orthopic liver transplantation and blood withdrawal

	Univariate Analysis		***Multivariate Analysis***	
Variable	***P* value**	**OR (95% CI)**	***P* value**	**OR (95% CI)**
Age	0.919	0.997 (0.942 – 1.055)		
Gender patient	0.339	1.704 (0.572 – 5.081)		
Gender donor	0.938	0.959 (0.332 – 2.770)		
Time between OLT and BW	0.434	0.996 (0.985 – 1.007)		
ITBL	0.388	1.902 (0.441 – 8.194)		
HCC pre-OLT	0.353	0.581 (0.185 – 1.828)		
Tacrolimus intake	0.207	0.296 (0.045 – 1.965)		
ALT	0.091	0.980 (0.957 – 1.003)		
AST	0.138	0.973 (0.938 – 1.009)		
GGT	0.892	1.000 (0.997 – 1.003)		
Sphingosine-1-phosphate	0.749	0.999 (0.993 – 1.005)		
Sphinganine	0.932	0.992 (0.823 – 1.195)		
Sphinganine-1-phosphate	0.449	0.993 (0.973 – 1.012)		
C18Cer	0.475	1.009 (0.985 – 1.033)	0.072	0.973 (0.945 – 1.002)
C18:1Cer	0.571	1.016 (0.962 – 1.072)		
C20Cer	0.225	1.017 (0.990 – 1.045)		

C24Cer	0.048	1.001 (1.000 – 1.002)	0.039	1.001 (1.000 – 1.002)
C18DHC	0.073	1.084 (0.992 – 1.183)	0.035	1.113 (1.009 – 1.269)
C24:1DHC	0.113	1.018 (0.994 – 1.043)		

Abbreviations: OR, odds-ratio; CI, confidence interval; BW, blood withdrawal; ITBL, ischemic type biliary lesions; HCC, hepatocellular carcinoma; OLT, orthopic liver transplantation; ALT, alanine transferase; AST, aspartate transferase; GGT, gamma glutamyl transferase; Cer, ceramide; DHC, dihydroceramide. Missing data: GGT level was missing in 1 patient, unknown donors' gender in 37 patients; missing.

Suppl. Table S2. Multivariate analysis for history of liver graft rejection including AB0-matching.

	Univariate Analysis		***Multivariate Analysis***	
Variable	***P* value**	**OR (95% CI)**	***P* value**	**OR (95% CI)**
Age	0.850	1.006 (0.950 – 1.065)		
Gender patient	0.271	1.874 (0.613 – 5.731)		
Gender donor	0.834	0.889 (0.296 – 2.668)		
AB0-matching	0.937	1.314E+05 (0.000 – 6. 41E+131)		
ITBL	0.289	2.242 (0.504 – 9.980)		
HCC pre-OLT	0.202	0.448 (0.131 – 1.537)		
Tacrolimus intake	0.204	0.292 (0.044 – 1.949)		
ALT	0.126	0.981 (0.958 – 1.005)		
AST	0.160	0.974 (0.939 – 1.011)		
GGT	0.401	0.998 (0.994 – 1.002)		
Sphingosine-1-phosphate	0.636	0.998 (0.991 – 1.005)		
Sphinganine	0.964	0.995 (0.813 – 1.219)		
Sphinganine-1-phosphate	0.360	0.990 (0.970 – 1.011)		
C18Cer	0.433	1.010 (0.986 – 1.035)	0.096	0.975 (0.945 – 1.005)
C18:1Cer	0.700	1.011 (0.957 – 1.068)		
C20Cer	0.209	1.018 (0.990 – 1.046)		

C24Cer	0.065	1.008 (1.000 – 1.002)	0.062	1.001 (1.000 – 1.002)
C18DHC	0.072	1.091 (0.992 – 1.200)	0.040	1.140 (1.006 – 1.293)
C24:1DHC	0.134	1.019 (0.994 – 1.046)		

Abbreviations: OR, odds-ratio; CI, confidence interval; ITBL, ischemic type biliary lesions; HCC, hepatocellular carcinoma; OLT, orthopic liver transplantation; ALT, alanine transferase; AST, aspartate transferase; GGT, gamma glutamyl transferase; Cer, ceramide; DHC, dihydroceramide. Missing data: AB0-matching was missing in 25 cases, GGT level was missing in 1 patient, unknown donors' gender in 37 patients; missing.

Suppl. Table S3. Multivariate analysis for C24Cer including age, gender patient, graft's age, gender donor, time between OLT and BW, ITBL, HCC pre-OLT, tacrolimus through level, history of liver graft rejection, ALT and GGT.

	Univariate Analysis		***Multivariate Analysis***	
Variable	***P* value**	**Beta/SD(beta)/Std.beta**	***P* value**	**Beta/SD(beta)/Std.beta**
Age	0.209	14.873/11.748/0.144		
Gender patient	0.556	-143.614/242.799/-0.069		
Graft's age	0.092	-9.179/5.373/-0.196		
Gender donor	0.803	-58.477/233.314/-0.030		
Time between OLT/BW	0.347	-2.251/2.380/-0.110		
ITBL	0.987	5.703/362.093/0.002		
HCC pre-OLT	0.862	-40.318/230.824/-0.020		
Tacrolimus through level	0.089	60.187/34.981/0.202	0.057	59.590/30.881/0.200
Graft rejection	0.099	492.544/295.273/0.188	0.039	567.7199/271.000/0.217
ALT	0.840	-1.281/6.323/-0.027		
GGT	0.172	-0.952/0.691/-0.186		

Abbreviations: BW, blood withdrawal; ITBL, ischemic type biliary lesions; HCC, hepatocellular carcinoma; OLT, orthopic liver transplantation; ALT, alanine transferase; GGT, gamma glutamyl transferase. Missing data: GGT level was missing in 1 patient, unknown donors' gender in 37 patients.

Suppl. Table S4. Multivariate analysis for C24:1Cer including age, gender patient, graft's age, gender donor, HCC pre-OLT, tacrolimus through level, AST, ALT and GGT.

	Univariate Analysis		***Multivariate Analysis***	
Variable	***P* value**	**Beta/SD(beta)/Std.beta**	***P* value**	**Beta/SD(beta)/Std.beta**
Age	0.084	5.304/3.038/0.176	0.039	6.042/2.887/0.200
Gender patient	0.936	-5.367/66.241/-0.009		
Graft's age	0.037	-3-297/1.559/-0.229	0.004	-0.416/1.380/-0.286
Gender donor	0.117	101.041/63.862/0.171		
HCC pre-OLT	0.935	-5.214/63.300/-0.009		
Tacrolimus through level	0.020	22.922/9.640/0.246	0.004	25.684/8.703/0.276
AST	0.625	-1.581/3.222/-0.073		
ALT	0.929	-0.204/2.274/-0.014		
GGT	0.560	0.105/0.179/0.065		

Abbreviations: HCC, hepatocellular carcinoma; OLT, orthopic liver transplantation; AST, aspartate transferase; ALT, alanine transferase; GGT, gamma glutamyl transferase. Missing data: GGT level was missing in 1 patient, unknown donors' gender in 37 patients.

Suppl. Table S5. Multivariate analysis for C24:1DHC including age, gender patient, graft’s age, gender donor, HCC pre-OLT, tacrolimus through level, AST, ALT and GGT.

	Univariate Analysis		***Multivariate Analysis***	
Variable	***P* value**	**Beta/SD(beta)/Std.beta**	***P* value**	**Beta/SD(beta)/Std.beta**
Age	0.356	0.321/0.345/0.098		
Gender patient	0.315	7.606/7.530/0.111		
Graft’s age	0.743	0.058/0.177/0.037		
Gender donor	0.523	4.655/7.259/0.073		
HCC pre-OLT	0.435	-5.643/7.195/-0.086		
Tacrolimus through level	0.089	1.883/1.096/0.187	0.047	2.018/1.001/0.201
AST	0.141	0.544/0.366/0.232		
ALT	0.494	-0.178/0.259/-0.113		
GGT	0.827	0.005/0.020/0.026		

<u>Abbreviations:</u> HCC, hepatocellular carcinoma; OLT, orthopic liver transplantation; AST, aspartate transferase; ALT, alanine transferase; GGT, gamma glutamyl transferase. <u>Missing data:</u> GGT level was missing in 1 patient, unknown donors’ gender in 37 patients.

Suppl. Table S6. Precursor-to-product ion transitions used for quantification of sphingolipids and corresponding mass spectrometric parameters.

Analyte	Precursor ion Q1 *[m/z]*	Product ion Q3 *[m/z]*	CE [V]	DP [V]	CXP [V]	Internal Standard
Sphingosine	300.3	282.1	17	42	20	Sphingosine-d7
Sphinganine	302.3	252.0	25	42	20	Sphinganine-d7
Sphingosin-1-phosphate	380.2	264.2	23	54	8	Sphingosin-1-phosphate-d7
Sphinganine-1-phosphate	382.3	284.1	19	58	20	Sphingosin-1-phosphate-d7
C14:0 Cer	510.5	264.2	40	60	15	C17:0 Cer
C16:0 Cer	538.5	264.2	40	60	18	C16:0 Cer-d31
C18:0 Cer	566.5	264.2	44	60	18	C18:0 Cer-d3
C20:0 Cer	594.6	264.2	45	60	19	C18:0 Cer-d3
C24:1 Cer	648.6	264.2	48	70	15	C24:0 Cer-d4
C24:0 Cer	650.6	264.2	47	75	19	C24:0 Cer-d4
C16:0 DHC	540.5	284.2	42	93	15	C18:0 DHC-d3
C18:0 DHC	568.6	284.2	45	85	17	C18:0 DHC-d3
C24:1 DHC	650.6	284.2	49	95	17	C24:0 Cer-d4
C24:0 DHC	652.7	284.2	47	95	15	C24:0 Cer-d4

Abbreviations: CE=collision energy, DP=declustering potential, CXP=collision cell exit potential.

Supplementary Figures

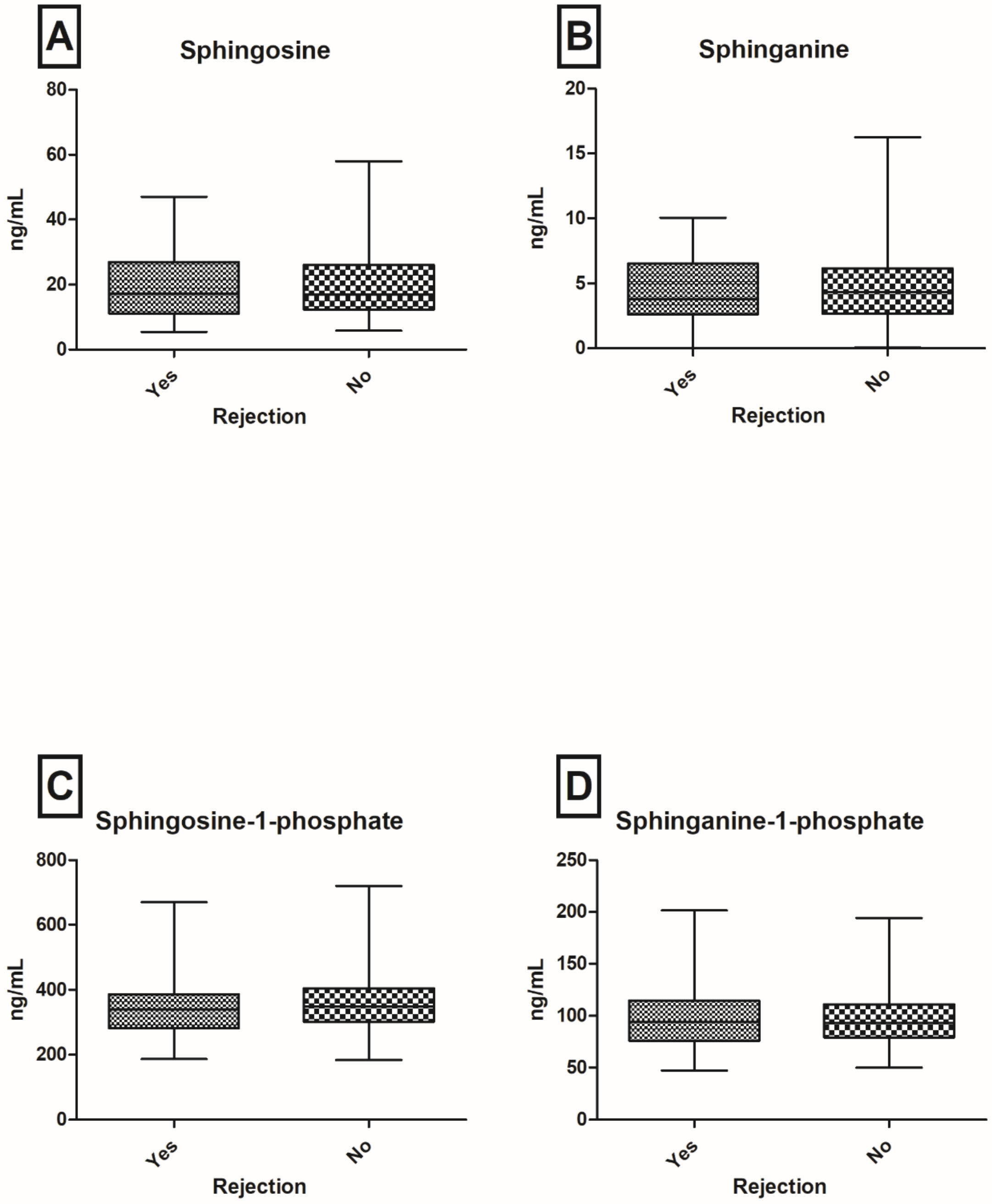

Suppl.Fig.S1. Serum sphingosine, sphinganine and their phosphate derivates in post-OLT patients with history of graft rejection.

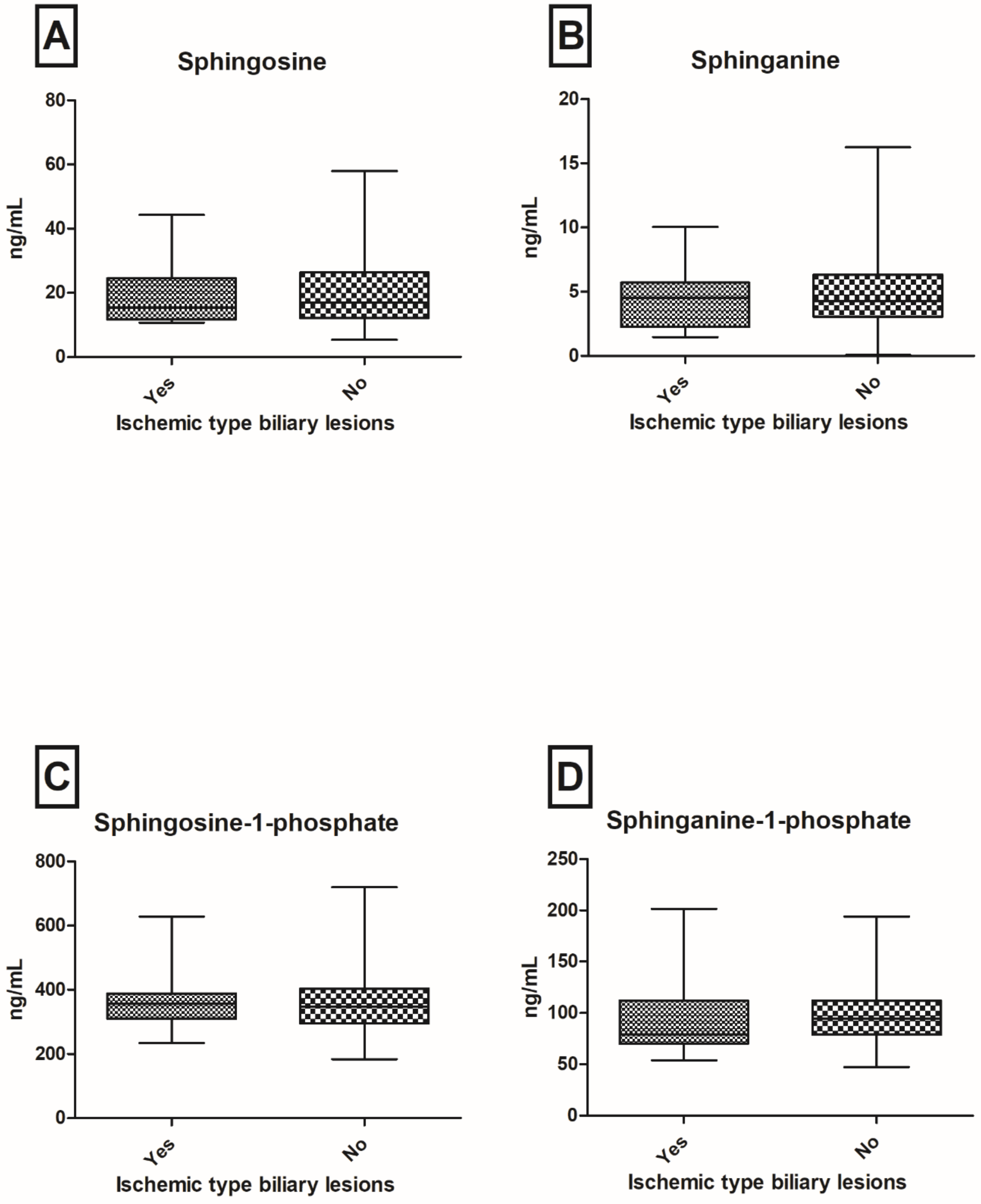

Suppl. Fig. S2. Serum sphingosine, sphinganine and their phosphate derivates in post-OLT patients with history of ischemic type biliary lesions.

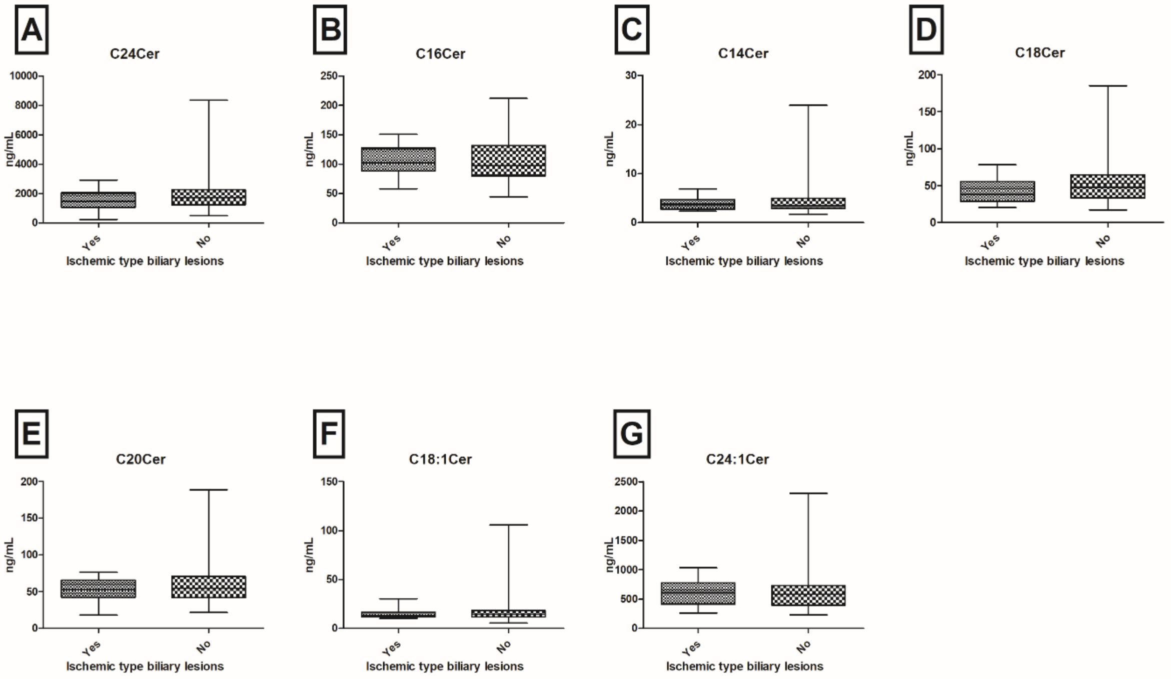

Suppl. Fig. S3. Serum ceramides in post-OLT patients with history of ischemic type biliary lesions.

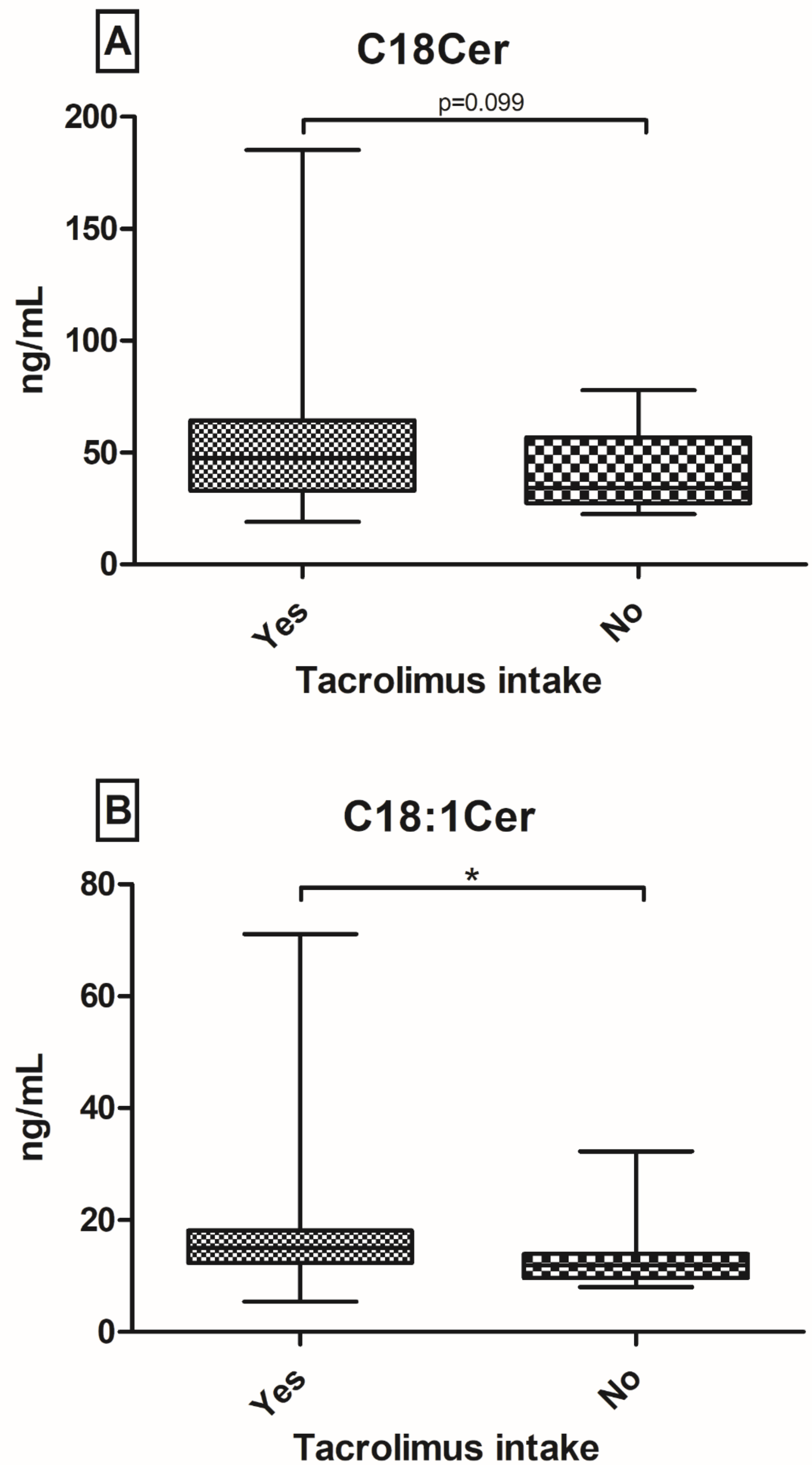

Suppl. Fig. S4 Serum ceramides (Cer) in propensity score matched post-OLT patients in regard to tacrolimus intake. C18Cer (A) and C18:1Cer (B) are up-regulated in patients with current tacrolimus intake, compared to no tacrolimus intake ("*"=$p<0.05$).
